# Relationship between Systemic Inflammatory Markers, GLUT1 Expression, and Maximum 18F-Fluorodeoxyglucose Uptake in Non-Small Cell Lung Carcinoma and Their Prognostic Significance

**DOI:** 10.3390/diagnostics13061013

**Published:** 2023-03-07

**Authors:** Sonya Youngju Park, Deog-Gon Cho, Byoung-Yong Shim, Uiju Cho

**Affiliations:** 1 Department of Nuclear Medicine, Seoul St. Mary’s Hospital, College of Medicine, The Catholic University of Korea, Seoul 06591, Republic of Korea; 2 Department of Thoracic Surgery, St. Vincent’s Hospital, College of Medicine, The Catholic University of Korea, Seoul 06591, Republic of Korea; 3 Division of Medical Oncology, Department of Internal Medicine, St. Vincent’s Hospital, College of Medicine, The Catholic University of Korea, Seoul 06591, Republic of Korea; 4 Department of Pathology, St. Vincent’s Hospital, College of Medicine, The Catholic University of Korea, Seoul 06591, Republic of Korea

**Keywords:** inflammation, monocyte, GLUT1, PET-CT scan, non-small cell lung cancer, prognosis, survival

## Abstract

Background: Factors involved in inflammation and cancer interact in various ways with each other, and biomarkers of systemic inflammation may have a prognostic value in cancer. Glucose transporter 1 (GLUT1) plays a pivotal role in glucose transport and metabolism and it is aberrantly expressed in various cancer types. We evaluated the differential expression of GLUT1, along with 18F-fluorodeoxyglucose positron emission tomography (FDG-PET) in non-small-cell lung cancer (NSCLC), and then analyzed their prognostic significance. Methods: A total of 163 patients with resectable NSCLC were included in this study. Tumor sections were immunohistochemically stained for GLUT1 and GLUT3. Maximum standardized uptake value (SUV_max_) was measured by preoperative FDG-PET, and neutrophil–lymphocyte ratio (NLR), platelet–lymphocyte ratio (PLR), and lymphocyte–monocyte ratio (LMR) were derived from pretreatment blood count. Results: GLUT1 and GLUT3 was positively expressed in 74.8% and 6.1% of the NSCLC tissues, respectively. GLUT1 expression was significantly correlated with squamous cell carcinoma histology, poor differentiation, high pathologic stage, old age, male, smoking, and high SUV_max_ (>7) (all *p* < 0.05). The squamous cell carcinoma and smoker group also showed significantly higher SUV_max_ (both *p* < 0.001). Systemic inflammation markers, including NLR, PLR, and LMR, were positively correlated with high SUV_max_ (all *p* < 0.05). High GLUT1 expression, high SUV_max_, high NLR, and low LMR, were significantly associated with poor overall survival in patients with NSCLC. However, in the multivariate survival analysis, LMR was an independent prognostic factor overall (HR 1.86, 95% CI 1.05–3.3) and for the stage I/II cohort (HR 2.3, 95% CI 1.24–4.3) (all *p* < 0.05). Conclusions: Systemic inflammatory markers—NLR, PLR, and LMR are strongly correlated with the SUV_max_ and are indicators of aggressive tumor behavior. Specifically, LMR is a promising prognostic biomarker in NSCLC patients.

## 1. Introduction

Despite recent therapeutic advances, the prognosis of non-small-cell lung cancer (NSCLC) is still poor, and it is a leading cause of cancer death worldwide [1]. The diverse nature of this cancer contributes to its high mortality rate [2]. Therefore, understanding the biology of NSCLC and its patient stratification is important for designing optimal treatments.

The hallmarks of cancer are distinctive capabilities that make tumor growth and metastasis possible [3]. They include sustaining proliferative signaling, evading growth suppressors, activating invasion and metastasis, enabling replicative immortality, inducing angiogenesis, and resisting cell death. In addition to these classic hallmarks, two new hallmarks are emerging: reprogramming energy metabolism and avoiding immune destruction [3]. Cancer cells’ ability to reprogram their glucose metabolism was first discovered by Otto Warburg and is called the Warburg Effect [4]. The Warburg Effect is characterized by increased glycolysis in cancer cells, even in the presence of oxygen. This metabolic switch in cancer cells is accomplished in part by upregulating glucose transporters, especially glucose transporter-1 (GLUT1), and increases glucose influx into the cytoplasm [4].

GLUT1 is a member of a glucose transporter family (GLUT), which transports glucose across the cell membrane. Seven glucose transporters in this family have been discovered [5]. GLUT1 is overexpressed in various cancers, including lung, colorectal, and breast cancers [6,7,8,9]. Increased uptake and utilization of glucose have also been observed by noninvasively visualizing glucose uptake using a radiolabeled analog of glucose (18F-fluorodeoxyglucose, FDG) and positron emission tomography (PET). FDG-PET parameters are tumor glucose metabolic markers. Typically, the maximum standardized uptake value (SUV_max_) has been associated with the progression, metastasis, and prognosis of various cancer types, including lung cancer [10,11,12,13,14,15].

Another emerging hallmark of cancer is its evasion of immune responses [3]. Cancer cells are suspected of having mechanisms that help them evade immunological monitoring or limit the extent of immunological killing [3,16,17]. Recent studies have reported new findings regarding this ability of cancer cells, as well as the interaction between tumor cells and immune cells in the tumor microenvironment and the relationship between tumor cells and systemic inflammatory responses [18,19]. One of the human body’s immune responses to cancer is changing the populations of circulating leukocytes and platelets. Similar to bacterial or virus infections, patients with cancer often develop thrombocytosis or neutrophilia, and these immune responses are reflected by systemic inflammatory markers such as the neutrophil–lymphocyte ratio (NLR), the platelet–lymphocyte ratio (PLR), and the lymphocyte–monocyte ratio (LMR) [20,21]. Interestingly, high NLR and PLR are increasingly reported to be associated with poor prognosis in different cancers [22,23,24,25,26,27]. Although LMR has been reported as a prognostic marker for several cancers, including lung cancer, there is currently a lack of sufficient evidence [28,29]. While it is not a novel concept, the association of systemic inflammation with cancer has increasingly become the subject of current research [30].

The clinical meaning of tumor metabolic activity and systemic inflammation in lung cancer has been extensively investigated in previous studies. However, the relationship of these key features has not been clearly elucidated, and only few studies have evaluated these hallmarks in NSCLC rather than in small-cell carcinoma [15]. Therefore, herein, we evaluated 18-F-FDG PET-CT, GLUT1 expression, NLR, PLR, and LMR, among other indicators of tumor metabolic activity and systemic inflammation, for their association with NSCLC and compared their prognostic values. Since these factors can be easily examined in a clinical context, our findings are valuable for the therapeutic management of NSCLC.

## 2. Methods

### 2.1. Patients

A total of 163 patients diagnosed with NSCLC in St. Vincent’s Hospital between 2006 and 2016 were included in the study. All patients underwent surgical resection or excisional biopsy. The patients’ clinical information and pathological data were obtained from hospital medical records. The patients were classified by cancer stage at the time of the surgery, and patients diagnosed before the announcement of staging guidelines of the 7th American Joint Committee on Cancer were restaged according to the 7th edition of the TNM classification of malignant tumors [31]. Pure ground-glass lesions were excluded from the study. Patients did not receive neoadjuvant chemotherapy or radiotherapy.

Using patients’ medical reports, we recorded their differential white blood cell (WBC) counts, taken within one month of surgery or excisional biopsy as part of a preoperative workup. Inflammatory markers were defined as follows: NLR (absolute neutrophil count/absolute lymphocyte count), PLR (absolute platelet count/absolute lymphocyte count), and LMR (absolute lymphocyte count/absolute monocyte count).

The study was conducted according to the World Medical Association Declaration of Helsinki and the study protocol was approved by the Institutional Review Board (IRB) of St. Vincent’s Hospital at The Catholic University of Korea (IRB No. VC20SISI0017). Written informed consent was obtained from all patients.

### 2.2. FDG/PET-CT Protocol and Image Analysis

After the patients fasted for a minimum of six hours, 3.7–5.5 MBq/kg of 18F-FDG was injected intravenously. None of the patients had a blood glucose level exceeding 130 mg/dL pre-injection. No contrast agent was given. Studies were acquired using a combined PET/CT in-line system (Biograph TruePoint, Siemens Medical Solutions, Knoxville, TN, USA), for 2–3 min per bed position.

FDG PET/CT images were reviewed by an expert nuclear medicine physician (S.Y.P.). FDG avidity was defined as showing discrete uptake exceeding the background soft tissue visual. The maximum standardized uptake value (SUV_max_) of the primary lung lesion was calculated from the injected dose and body weight. When there were multifocal lesions, the region of interest was drawn for the largest lesion. The SUV_max_ was calculated as follows:

SUV = *C*/(*Di*/*W*)

*C* is the decay-corrected tracer tissue concentration (kBq/mL), *Di* is the injected dose (MBq), and *W* is the body weight (kg).

### 2.3. Immunohistochemistry

For the construction of tissue microarrays (TMAs), the most representative areas were identified on a slide stained with hematoxylin and eosin and marked by a pathologist (U.C). One core measuring 5.0 mm in diameter was obtained and arrayed onto a paraffin block. Each sample was tested using primary antibodies for GLUT1 (Abcam, Cambridge, UK; diluted 1:200) and GLUT3 (Abcam; diluted 1:200). Briefly, 4-µm sections were deparaffinized in xylene and then rehydrated through a graded ethanol series. Slides were loaded into a BenchMark XT automated slide stainer (Ventana Medical Systems, Inc., Oro Valley, AZ, USA) and then incubated for 16 min at 37 °C with each primary antibody. Immunoreactivity was detected using an ultraView Universal DAB detection kit (Ventana Medical Systems, Inc.) and 3,3′-diaminobenzidine, followed by counterstaining with hematoxylin and a bluing agent.

An expert pathologist (U.C.) performed immunohistochemical assessments. For the evaluation of immunoreactivity in tumor cells, a dichotomized scoring system was used as follows: GLUT1 and GLUT3 positivity was determined if ≥10% of tumor cells demonstrated either membranous and/or cytoplasmic staining.

### 2.4. Statistical Analysis

We applied chi-squared tests to compare categorical variables. Overall survival (OS) was defined from the date of the initial diagnosis to the date of death. Progression-free survival was defined from the date of the initial diagnosis to the data of progression. Survival estimates were analyzed based on the Kaplan–Meier method and compared using the log-rank test and univariate Cox proportional hazard regression analysis. Factors that were significant according to univariate analysis or factors that were considered clinically important were subjected to Cox proportional hazards regression multivariate analysis.

The ideal cutoff value for the FDG-PET SUV_max_ was determined via receiver operating characteristic (ROC) curve analysis. A score closest to the point of maximum sensitivity and specificity was selected, leading to the largest group of tumors that correctly predicted the survival event. Median values were used as cutoffs for NLR, PLR, and LMR. A nomogram for possible prognostic factors used the R packages survival and rms. The performance of the nomogram for predicting survival was evaluated with Harrell’s concordance index (C-index). The bootstrapping method was used for internal validation of the nomogram. A two-sided *p* value < 0.05 was considered significant. R software (version 4.2.2) was used for the statistical analyses.

## 3. Results

### 3.1. Study Population: Demographic and Clinical Features

This study cohort included 105 adenocarcinoma (105/163, 64.4%), 49 squamous cell carcinoma (49/163, 30.1%), and nine other histological types of cancer (e.g., large cell carcinoma and pleomorphic carcinoma: 9/163; 5.5%) cases. The mean age was 64.8 years (ranging from 36 to 82), and males outnumbered females (male/female ratio: 2.13). Records showed that 43.6% of patients involved in the study had never smoked, and patients were distributed among each discrete cancer staging category as follows: IA: 21.5%, IB: 16.6%, IIA: 16.6%, IIB: 8.6%, IIIA: 18.4%, IIIB: 2.5%, and IV: 16.0%. About two-thirds (103/163, 63.2%) of patients received adjuvant therapy, including chemotherapy, radiation therapy, combined chemoradiation therapy, or tyrosine kinase inhibitor therapy, while others (60/163, 36.8%) received only surgery.

### 3.2. GLUT1 and GLUT3 Expression and FDG-PET SUV_max_ Characterization

**Figure 1:**
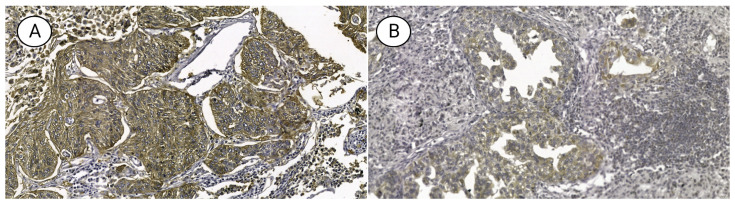
Micrograph of non-small cell lung cancer-expressing (**A**) GLUT1 and (**B**) GLUT3 (×400).

GLUT1 and GLUT3 were positively expressed in 74.8% and 6.1% of patients, respectively. GLUT1 expression was significantly correlated with male patients, smokers, and squamous cell carcinomas with poor histologic differentiation (all *p* < 0.05) (Figure 1). The average FDG-PET SUV_max_ was 7.1 ± 3.8, and it was significantly higher in the GLUT1-positive compared to the GLUT1-negative group (*p* < 0.001) (Table 1). FDG-PET SUV_max_ values ranged from 0.6 to 19.6 (median 5.9), and the mean was 6.4 (standard deviation 3.83). On the other hand, GLUT3 expression did not correlate with any clinicopathologic features (all *p* > 0.05). A high SUV_max_ value (>7) also significantly correlated with male patients, smokers, squamous cell carcinomas with poor differentiation, as well as patients in advanced T stage or AJCC stage (all *p* < 0.05) (Table 1).

### 3.3. NLR and PLR Characterization and Their Association with Clinicopathologic Characteristics

Among all patients with NSCLC, the mean white blood cell count was 7.7 ± 2.6 × 10^6^/mL, and the mean NLR, PLR, and LMR were 2.8 ± 3.0, 4.0 ± 1.8, and 133.8 ± 84.8, respectively. Patients with elevated NLR had a more advanced AJCC stage, and mean FDG-PET SUV_max_ and PLR were higher in the high NLR group (all *p* < 0.05). The mean LMR was significantly lower in the high NLR group (*p* < 0.001). Elevated PLR was associated with high FDG-PET SUV_max_ and high LMR (all *p* < 0.05). Elevated LMR was correlated with females and a less advanced AJCC stage (all *p* < 0.05). The mean FDG-PET SUV_max_, NLR, and PLR were significantly lower in the high LMR group (all *p* < 0.05) (Table 2).

### 3.4. Correlation of Metabolic Markers and Systemic Inflammatory Markers in Patients with Stage I and II NSCLC

Additionally, we performed a subgroup analysis in 103 patients with AJCC Stage I and II low-stage groups. As among all patients with NSCLC, GLUT1 expression was correlated with high FDG-PET SUV_max_ (6.8 ± 3.6 vs. 3.3 ± 2.5, *p* < 0.001), but GLUT3 expression showed no correlation with any other markers. NLR and PLR were also positively corelated with the FDG-PET SUV_max_ (all *p* < 0.05). However, LMR had no correlation with FDG-PET SUV_max_ in this subgroup (*p* = 0.498).

### 3.5. Survival Analysis

The follow-up period ranged from 1 to 139.9 months (median 5.8 months). Ninety patients (55.2%) died during the follow-up, and the median OS was 55.0 months. The survival rates at 2 and 5 years after diagnosis were 71.2% (standard error 3.6%) and 51.7% (standard error 4.0%), respectively. After surgery, 26.4% of the patients received adjuvant chemotherapy, radiotherapy, or chemoradiotherapy.

**Figure 2:**
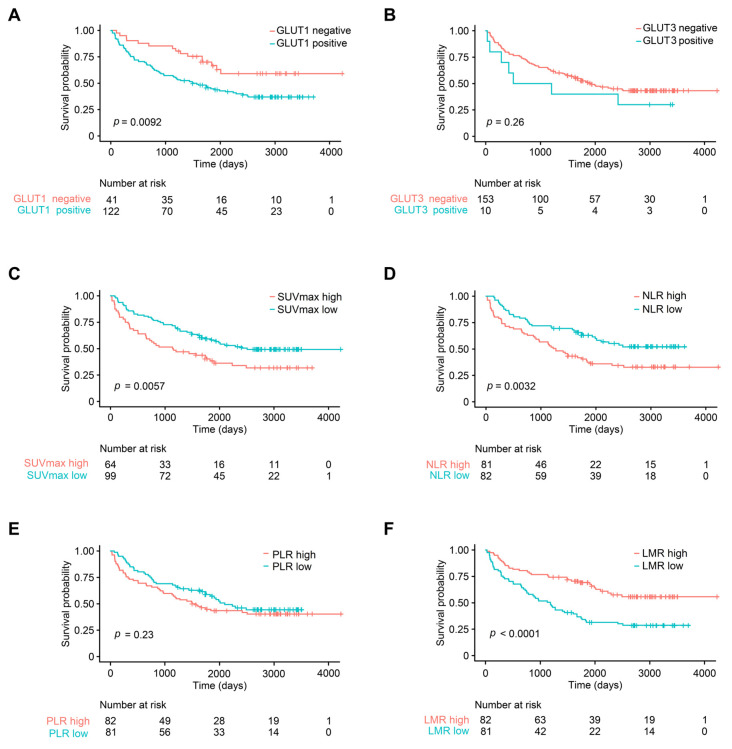
Kaplan–Meier survival curves of different parameters in patients with non-small cell carcinoma. (**A**) GLUT1 expression, (**B**) GLUT3 expression, (**C**) FDG-PET SUV_max_, (**D**) neutrophil–lymphocyte ratio (NLR), (**E**) platelet–lymphocyte ratio (PLR), and (**F**) lymphocyte–monocyte ratio (LMR). FDG-PET SUV_max_, fluoro-D-glucose-positron emission tomography maximum uptake value.

**Figure 3:**
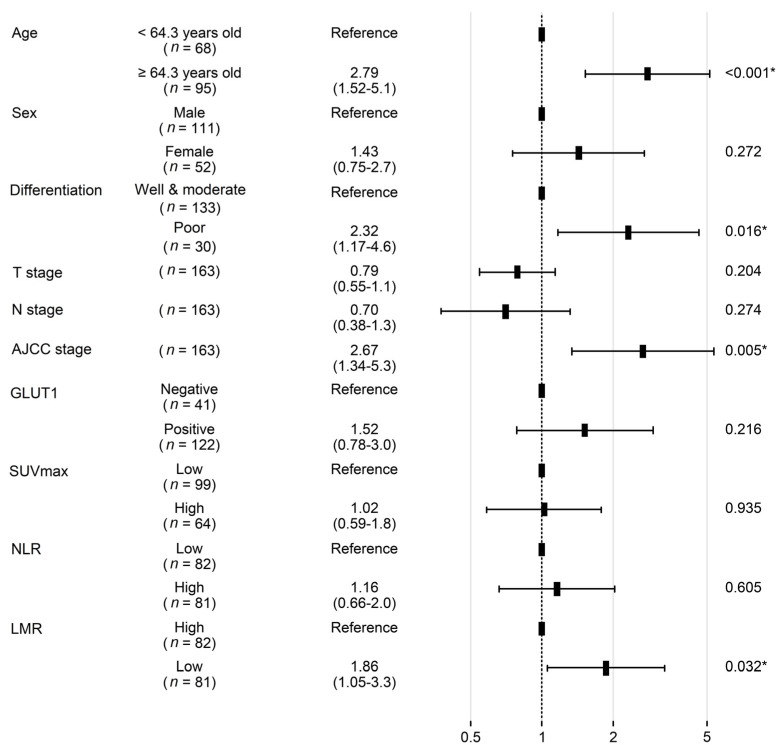
Forest plot showing hazard ratios obtained by multivariate Cox regression for overall survival in patients with non-small cell carcinoma. SUV_max_, fluoro-D-glucose-positron emission tomography maximum uptake value; NLR—neutrophil–lymphocyte ratio; LMR—lymphocyte–monocyte ratio. * Indicates factors with significant *p* values.

In the univariate analysis, poor tumor differentiation, high GLUT1 expression, high SUV_max_, high NLR, low LMR, and advanced T, N, and AJCC stage were associated with worse OS (all *p* < 0.05) (Figure 2). However, age, sex, smoking history, squamous cell carcinoma histology, lymphovascular invasion, GLUT3 expression, and high PLR demonstrated no prognostic significance (all *p* > 0.05). In a multivariate analysis, old age, AJCC stage, and LMR were independent prognostic factors that were associated with OS (all *p* < 0.05) (Figure 3).

Survival analysis was then performed in patients with AJCC stage I, II, and III NSCLC and in patients with AJCC stage IV NSCLCL, i.e., with metastasis. In the patient with AJCC stage I, II, and III NSCLC, old age, poor histologic differentiation, AJCC stage, and LMR were independent prognostic factors with regard to OS (all *p* < 0.05) (Table 3). Progression-free survival was analyzed in the patient with AJCC stage IV NSCLC, and none of the clinicopathologic, metabolic, or systemic inflammatory markers were associated with progression (all *p* > 0.05)

**Figure 4:**
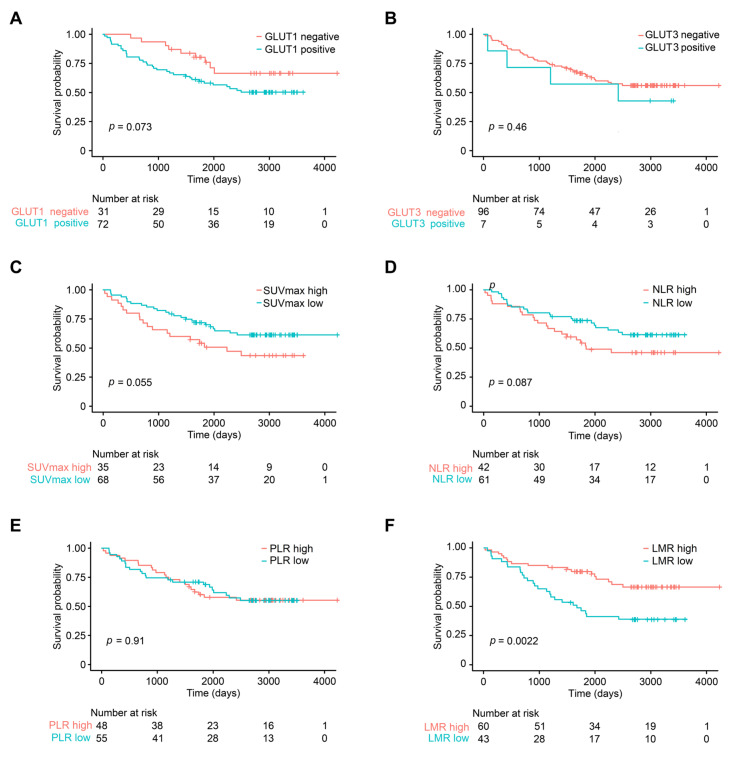
Kaplan–Meier survival curves of different parameters in patients with stage I/II non-small cell carcinoma. (**A**) GLUT1 expression, (**B**) GLUT3 expression, (**C**) FDG-PET SUV_max_, (**D**) neutrophil–lymphocyte ratio (NLR), (**E**) platelet–lymphocyte ratio (PLR), and (**F**) lymphocyte–monocyte ratio (LMR). FDG-PET SUV_max_, fluoro-D-glucose-positron emission tomography maximum uptake value.

**Figure 5:**
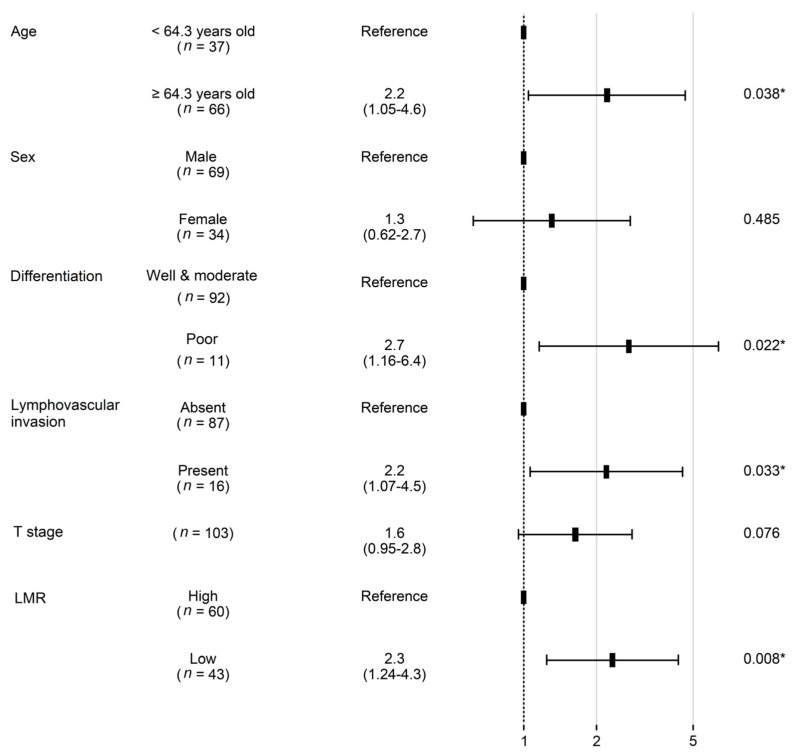
Forest plot showing hazard ratios obtained by multivariate Cox regression for overall survival in patients with stage I/II non-small cell carcinoma. LMR—lymphocyte–monocyte ratio. * Indicates factors with significant *p* values.

In the subgroup of patients with stage I and II NSCLC, 42.7% of the patients died during the follow-up period, and the median OS was 65.3 months (range 1 to 139.9 months). The 2-year survival rate was 81.6% (standard error 3.8%) and the 5-year survival rate was 65.8% (standard error 4.7%). In this group, histological differentiation, lymphovascular invasion, old age, T stage, and LMR were associated with OS (all *p* < 0.05) (Figure 4). The 2-year survival rate of patients with low LMR was 74.4%, which was significantly lower when compared to that of the high-LMR group, which was 86.7% (*p* = 0.002). GLUT1 expression, GLUT3 expression, squamous cell carcinoma histology, sex, high SVU_max_, N stage, NLR, and PLR were not significant prognostic factors in stage I and II group (all *p* > 0.05). According to multivariate Cox proportional hazards analysis, old age, poor differentiation, lymphovascular invasion, and low LMR remained independent prognostic factors associated with poor OS in patients with stage I/II NSCLC (all *p* < 0.05). Low LMR was a particularly poor prognostic factor, with a hazard ratio of 2.3 (95% CI 1.2–4.3, *p* = 0.008) (Figure 5).

### 3.6. Nomogram for Prediction of OS

**Figure 6:**
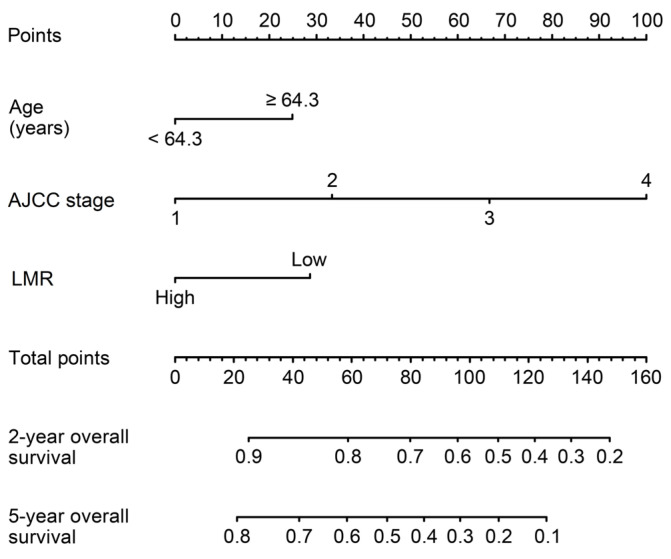
Nomogram for overall survival of patients with stage I/II non-small cell carcinoma. LMR—lymphocyte–monocyte ratio.

A prognostic nomogram of patients with NSCLC was established using a Cox regression model according to significant independent prognostic factors of OS (age, AJCC stage, and LMR). Each factor in the nomogram was assigned a weighted number of points. The sum of points for each patient was in accordance with a specific predicted 3- and 5-year OS. A nomogram predicting OS was also established in the group of patients with stage I and II NSCLC (Figure 6). Independent prognostic factors in this group—such as age, histologic differentiation, lymphovascular invasion, and LMR—were incorporated in the nomogram. The C-index of the multivariate prognostic model slightly improved from 0.75 (standard error 0.02) to 0.65 (standard error 0.04) when LMR was added to the model, which was developed based on age, histological differentiation, and lymphovascular invasion.

## 4. Discussion

In this paper, we investigated the clinical significance of 18-F-FDG PET-CT SUV_max_, NLR, PLR, LMR, and expression of GLUT1 and GLUT3, which can easily be measured in clinical settings, among various tumor metabolic activity and systemic inflammation markers, in patients with NSCLC. Our findings showed that GLUT1 expression, NLR, and LMR are prognostic factors predicting the OS of patients with NSCLC.

Changes in metabolic activity can be measured in vivo via metabolomics, magnetic resonance spectroscopy, PET, and stable isotope tracing [32]. In particular, 18-F-FDG PET-CT is not only used to diagnose malignant tumors of different cancers, but also reflects treatment response [33], while parameters such as SUV_max_, metabolic tumor volume, and total lesion glycolysis are associated with the prognosis of patients with cancer [10,11,12,13,14,34]. Previous findings suggest that the uptake of 18F-FDG has an independent prognostic value in patients newly diagnosed with NSCLC [35]. However, in our study, PET SUV_max_ was not a significant prognostic factor. FDG-PET parameters are related to and affected by different tumor markers or biomarkers such as CFLYRA21-1, NSE, SCC-ag, ki67, and p53 [36], and MTV is known to better reflect the prognosis of patients with cancer when compared to SUV_max_ [14]. In general, 18F-FDG-PET is a promising biomarker in cancer prognosis; however, its statistical significance as a prognostic factor has not been demonstrated. Thus, further studies must be conducted to confirm the power of 18F-FDG-PET as a prognostic factor [37].

We investigated the expression and clinical significance of two representative GLUT family proteins, GLUT1 and GLUT2. Only the expression of GLUT1, not GLUT3, was associated with a shorter OS. Interestingly, GLUT1 expression and SUV_max_ significantly differed according to the tumor histology and smoking history of patients with NSCLC. Herein, both GLUT1 expression and SUV_max_ were higher in patients who had squamous histology and who were smokers. These results are in line with those of previous studies [38]. In a previous study that analyzed differential gene expression in lung squamous cell carcinoma and adenocarcinoma using The Cancer Genome Atlas datasets, GLUT1 had the highest mRNA expression level among GLUT family proteins in squamous cell carcinoma. Similarly, GLUT1 overexpression was phenotypically and specifically linked to the squamous cell carcinoma subtype rather than the adenocarcinoma patient group [38]. Additionally, in a cell line study, squamous cell carcinoma was a more glycolysis-reliant histological phenotype than adenocarcinoma, and lung squamous cell carcinoma had higher 18F-FDG uptake than adenocarcinoma [38]. In a study by Koh et al., immunohistochemical evaluation of GLUT1 expression also demonstrated close association with a squamous phenotype, micropapillary/solid histology, lymphovascular invasion, and advanced pTNM stage in NSCLC [7]. Based on these previous findings and our data, we conclude that GLUT1 contributes to tumor aggressiveness, especially in squamous cells. GLUT1 immunohistochemical staining does not reflect the in vivo metabolic activity, but it can easily be conducted as an ancillary test during surgery or biopsies. We conclude that GLUT1 is an important indicator that can reflect the metabolic activity of NSCLC.

Analysis of the relationship between systemic inflammatory markers and other clinicopathological factors revealed interesting findings. Increases in neutrophil, platelet, and monocyte counts in relation to lymphocyte counts were generally correlated with an advanced stage, tumor aggressiveness, and markers of tumor metabolic activity, e.g., SUV_max_ and GLUT1 expression. NLR, PLR, and LMR were indicators of aggressive tumor behavior. 

In our study, NLR, PLR, and LMR correlated with SUV_max_, in agreement with previous studies. In one study of head and neck cancer, NLR was positively correlated with FDG-PET metabolic markers, including SUV_max_ [39]. In NSCLC, LMR and NLR showed a weak significant correlation with SUV_max_ [15]. In another study, SUV_max_ and LMR were independent prognostic factors in patients with stage IIIB-IV NSCLC. A novel score combining these two factors was developed and was useful for prognostication [39]. Some researchers have suggested that the underlying mechanism of such correlations is the nonspecific inflammatory response, which may reflect increased metabolism in the primary tumor [40]. Another hypothesis is related to tumor oxygenation and suggests that larger, poorly oxygenated tumors show increased FDG uptake and metabolism, which may then induce a systemic inflammatory response [15]. Our findings support such a relationship in which the pretreatment systemic inflammatory markers correlate positively with cancer’s FDG metabolism markers.

The association and interaction between systemic inflammatory markers and another metabolic marker, GLUT1 expression, remains to be unexplored. The direct relationship of NLR, PLR, LMR, and GLUT1 expression in NSCLC tissues has not been studied. In our data, these systemic inflammatory marker levels were not associated with GLUT1 expression status. However, one study showed the link between GLUT1 copy number and immune biomarkers of various immune cells, such as CD20, CD8A, and CD68 [41]. In this study, GLUT1 overexpression had a negative relationship with tumor-infiltrating T-cells but a positive relationship with tumor-infiltrating neutrophils and dendritic cells [41]. The researchers of this study hypothesized that GLUT1 influences the immune microenvironment with yet unrevealed mechanisms [41]. 

Based on our findings, LMR was an independent prognostic factor not only in the total patient group, but also in early stage (stage I and II) patient groups. Conventionally, treatment for patients with stage I or II NSCLC is surgical resection, but it is possible that resected early stage patients with adverse prognostic factors, such as lymphovascular invasion, would benefit more from adjuvant treatment than those without [42]. In such patients, risk stratification is fundamental for adequate adjuvant therapy. In addition to conventional pathologic high-risk factors, such as lymphovascular invasion and differentiation, using systemic inflammatory factors that do not require additional tests can greatly help in designing a treatment plan. When comparing nomograms with and without LMR, the inclusion of LMR showed an increase in the C-index value from 0.65 to 0.75, representing a more accurate prediction. In individual patients with NSCLC, low LMR adds 28.7 points to the total score—that is, a decrease of approximately 5.7%—for the 2-year survival rate.

The interaction between tumors and inflammation has been studied in various cancers, including NSCLC [43]. Inflammatory cells are now thought to promote cancer onset and progression through disruption of the anti-tumor immune system and regulation of the tumor microenvironment and epigenetic alterations [44]. Monocytes are inflammatory cells that produce reactive oxygen species, reactive nitrogen species, and other cytokines, promoting DNA mutation and eventually leading to tumor progression [45]. Monocytes can potentially differentiate into tumor-associated macrophages (TAM). TAM may promote cancer progression, metastasis, and immune evasion via angiogenesis, secreting epithelial and vascular endothelial growth factors, extracellular matrix remodeling, and upregulating PD-1 expression [46]. With this underlying mechanism, a relative increase in the monocyte to lymphocyte count—that is, lower LMR—may be associated with cancer prognosis. LMR has been demonstrated as a promising prognostic marker in various solid cancers, including NSCLC [29]. 

Our study was limited because EGFR mutation test results were unavailable for most patients in the cohort. Most administered adjuvant therapies were non-tyrosine kinase inhibitors (TKI) and were conventional, but a few patients who experienced cancer recurrence underwent EGFR testing and received TKI therapy. Such heterogeneity in treatment methods may have affected the survival data and is thus a limitation of this study. However, a review of medical records showed that only a small number of patients (five in total) underwent TKI therapy, suggesting that the potential effects on the results of this study would have been minimal.

## 5. Conclusions

In this study, we showed that markers of tumor metabolic activity—GLUT1 and SUV_max_—were positively correlated with neutrophil, platelet, and monocyte count increases in relation to lymphocyte count. GLUT1, NLR, and LMR are predictors of OS in NSCLC patients, helping to identify high-risk patients in need of close surveillance and adjuvant therapy. Further studies are warranted to investigate whether addition of these biomarkers to the current staging system could improve survival prediction, and hence support treatment decision making for patients with NSCLC.

## Figures and Tables

**Table 1:** Relationship between metabolic markers and clinicopathologic features in patients with non-small cell lung carcinoma.

	GLUT1			GLUT3			FDG-PET SUV_max_		
	Negative (*n* = 41)	Positive (*n* = 122)	*p*	Negative (*n* = 153)	Positive (*n* = 10)	*P*	Low (<7) (*n* = 99)	High (≥7) (*n* = 64)	*p*
Age (years)	63.2 ± 9.4	65.3 ± 10.1	0.236	64.6 ± 9.8	67.7 ± 11.9	0.337	63.7 ± 9.7	66.5 ± 10.1	0.082
Sex									
Male (*n* = 111, 68.1%)	17 (41.5%)	94 (77.0%)	<0.001	103 (67.3%)	8 (80%)	0.629	58 (58.6%)	53 (82.8%)	0.002
Female (*n* = 52, 31.9%)	24 (58.5%)	28 (23.0%)		50 (32.7%)	2 (20%)		41 (41.4%)	11 (17.2%)	
Smoking Status									
Never smoker (*n* = 71, 43.6%)	27 (65.9%)	44 (36.1%)	0.002	67 (43.8%)	4 (40%)	0.999	53 (53.5%)	18 (28.1%)	0.002
Ever smoker (*n* = 92, 56.4%)	14 (34.1%)	78 (63.9%)		86 (56.2%)	6 (60%)		46 (46.5%)	46 (71.9%)	
Histology									
Non-SqCC (*n* = 115 70.6%)	40 (97.6%)	75 (61.5%)	<0.001	106 (69.3%)	8 (80%)	0.719	80 (80.8%)	34 (53.1%)	<0.001
SqCC (*n* = 48, 29.4%)	1 (2.44%)	47 (38.5%)		47 (30.7%)	2 (20%)		19 (19.2%)	30 (46.9%)	
Tumor differentiation									
WD and MD (*n* = 133, 81.6%)	39 (95.1%)	94 (77.0%)	0.019	127 (83.0%)	6 (60%)	0.162	87 (87.9%)	46 (71.9%)	0.018
PD (*n* = 30, 18.4%)	2 (4.88%)	28 (23.0%)		26 (17.0%)	4 (40%)		12 (12.1%)	18 (28.1%)	
Lymphovascular invasion									
Absent (*n* = 125, 76.7%)	34 (82.9%)	91 (74.6%)	0.380	117 (76.5%)	8 (80.0%)	0.999	77 (77.8%)	48 (75.0%)	0.826
Present (*n* = 38, 23.3%)	7 (17.1%)	31 (25.4%)		36 (23.5%)	2 (20.0%)		22 (22.2%)	16 (25.0%)	
T stage *									
1 (*n* = 50, 30.7%)	18 (46.2%)	32 (32.3%)	0.129	50 (38.2%)	0	0.164	40 (46.5%)	10 (19.2%)	0.007
2 (*n* = 66, 40.5%)	19 (48.7%)	47 (47.5%)		60 (45.8%)	6 (85.7%)		37 (43.0%)	29 (55.8%)	
3 (*n* = 18, 6.7%)	2 (5.1%)	16 (16.2%)		17 (13.0%)	1 (14.3%)		7 (8.1%)	11 (21.2%)	
4 (*n* = 4, 2.5%)	0	4 (4.0%)		4 (3.1%)	0		2 (2.3%)	2 (3.8%)	
N stage ^†^									
0 (*n* = 77, 47.2%)	26 (66.7%)	51 (52.0%)	0.139	72 (55.4%)	5 (71.4%)	0.558	52 (61.2%)	25 (48.1%)	0.37
1 (*n* = 30, 18.4%)	6 (15.4%)	24 (24.5%)		28 (21.5%)	2 (28.6%)		16 (18.8%)	14 (26.9%)	
2 (*n* = 29, 17.8%)	6 (15.4%)	23 (23.5%)		29 (22.3%)	0		16 (18.8%)	13 (25.0%)	
3 (*n* = 1, 0.6%)	1 (2.6%)	0		1 (0.8%)	0		1 (1.2%)	0	
AJCC Stage									
I (*n* = 62, 38.0%)	22 (53.7%)	40 (32.8%)	0.084	58 (37.9%)	4 (40.0%)	0.302	47 (47.5%)	15 (23.4%)	0.022
II (*n* = 41, 25.2%)	9 (22.0%)	32 (26.2%)		38 (24.8%)	3 (30.0%)		21 (21.2%)	20 (31.2%)	
III (*n* = 34, 20.9%)	7 (17.1%)	27 (22.1%)		34 (22.2%)	0		18 (18.2%)	16 (25.0%)	
IV (*n* = 30, 18.4%)	3 (7.3%)	23 (18.9%)		23 (15.0%)	3 (30.0%)		13 (13.1%)	13 (20.3%)	
SUV_max_	4.2 ± 2.9	7.1 ± 3.8	<0.001	6.3 ± 3.8	8.1 ± 3.5	0.157	-	-	-
NLR	2.9 ± 3.4	2.8 ± 2.9	0.915	2.9 ± 3.1	2.6 ± 1.3	0.579	2.5 ± 2.6	3.4 ± 3.4	0.091
PLR	120.8 ± 57.8	138.1 ± 91.9	0.16	131.8 ± 85.8	164.3 ± 64.5	0.241	119.2 ± 60.4	156.4 ± 109.4	0.015
LMR	4.4 ± 1.7	3.9 ± 1.8	0.148	4.1 ± 1.8	3.2 ± 1.1	0.12	4.2 ± 1.6	3.7 ± 2.1	0.085

SqCC, squamous cell carcinoma; WD, well differentiated; MD, moderately differentiated; PD, poorly differentiated; AJCC, American Joint Committee on Cancer; SUV_max_, maximum standardized uptake value; NLR, Neutrophil–lymphocyte ratio; PLR, Platelet–lymphocyte ratio; LMR, Lymphocyte–monocyte ratio. * Twenty-five patients have missing pathologic T stage data. ^†^ Twenty-six patients have missing pathologic N stage data.

**Table 2:** Relationship between systemic inflammatory markers and clinicopathologic features in patients with non-small cell lung carcinoma.

	NLR			PLR			LMR		
	Low (*n* = 82)	High (*n* = 81)	*p*	Low (*n* = 81)	High (*n* = 82)	*p*	Low (*n* = 81)	High (*n* = 82)	*p*
Age (years)	65.5 ± 9.3	64.0 ± 10.5	0.353	65.3 ± 9.1	64.3 ± 10.7	0.511	65.2 ± 10.5	64.3 ± 9.4	0.552
Sex									
Male (*n* = 111, 68.1%)	30 (36.6%)	59 (72.8%)	0.262	55 (67.9%)	56 (68.3%)	0.999	64 (79.0%)	47 (57.3%)	0.005
Female (*n* = 52, 31.9%)	52 (63.4%)	22 (27.2%)		26 (32.1%)	26 (31.7%)		17 (21.0%)	35 (42.7%)	
Smoking Status									
Never smoker (*n* = 71, 43.6%)	40 (48.8%)	31 (38.3%)	0.232	43 (53.1%)	33 (40.2%)	0.483	32 (39.5%)	39 (47.6%)	0.379
Ever smoker (*n* = 92, 56.4%)	42 (51.2%)	50 (61.7%)		38 (46.9%)	49 (59.8%)		49 (60.5%)	43 (52.4%	
Histology									
Non-SqCC (*n* = 115 70.6%)	62 (75.6%)	52 (64.2%)	0.156	52 (64.2%)	62 (75.6%)	0.156	54 (66.7%)	60 (73.2%)	0.463
SqCC (*n* = 48, 29.4%)	20 (24.4%)	29 (35.8%)		29 (35.8%)	20 (24.4%)		27 (33.3%)	22 (26.8%)	
Tumor differentiation									
WD and MD (*n* = 133, 81.6%)	69 (84.1%)	64 (79.0%)	0.52	69 (85.2%)	64 (78.0%)	0.33	62 (76.5%)	71 (86.6%)	0.146
PD (*n* = 30, 18.4%)	13 (15.9%)	17 (21.0%)		12 (14.8%)	18 (22.0%)		19 (23.5%)	11 (13.4%)	
Lymphovascular invasion									
Absent (*n* = 125, 76.7%)	66 (80.5%)	59 (72.8%)	0.332	65 (80.2%)	60 (73.2%)	0.377	63 (77.8%)	62 (75.6%)	0.887
Present (*n* = 38, 23.3%)	16 (19.5%)	22 (27.2%)		16 (19.8%)	22 (26.8%)		18 (22.2%)	20 (24.4%)	
T stage *									
1 (*n* = 50, 30.7%)	30 (40.0%)	20 (31.7%)	0.138	28 (38.4%)	22 (33.8%)	0.156	22 (34.9%)	28 (37.3%)	0.693
2 (*n* = 66, 40.5%)	38 (50.7%)	28 (44.4%)		34 (46.6%)	32 (49.2%)		30 (47.6%)	36 (48.0%)	
3 (*n* = 18, 6.7%)	6 ( 8.0%)	12 (19.0%)		11 (15.1%)	7 (10.8%)		8 (12.7%)	10 (13.3%)	
4 (*n* = 4, 2.5%)	1 ( 1.3%)	3 ( 4.8%)		0 ( 0.0%)	4 ( 6.2%)		3 ( 4.8%)	1 ( 1.3%)	
N stage ^†^									
0 (*n* = 77, 47.2%)	47 (62.7%)	30 (48.4%)	0.145	40 (55.6%)	37 (56.9%)	0.784	34 (54.0%)	43 (58.1%)	0.626
1 (*n* = 30, 18.4%)	16 (21.3%)	14 (22.6%)		15 (20.8%)	15 (23.1%)		13 (20.6%)	17 (23.0%)	
2 (*n* = 29, 17.8%)	11 (14.7%)	18 (29.0%)		16 (22.2%)	13 (20.0%)		15 (23.8%)	14 (18.9%)	
3 (*n* = 1, 0.6%)	1 ( 1.3%)	0 ( 0.0%)		1 ( 1.4%)	0 ( 0.0%)		1 ( 1.6%)	0 ( 0.0%)	
AJCC Stage									
I (*n* = 62, 38.0%)	40 (48.8%)	22 (27.2%)	0.012	34 (42.0%)	28 (34.1%)	0.207	26 (32.1%)	36 (43.9%)	0.032
II (*n* = 41, 25.2%)	21 (25.6%)	20 (24.7%)		21 (25.9%)	20 (24.4%)		17 (21.0%)	24 (29.3%)	
III (*n* = 34, 20.9%)	13 (15.9%)	21 (25.9%)		18 (22.2%)	16 (19.5%)		19 (23.5%)	15 (18.3%)	
IV (*n* = 30, 18.4%)	8 ( 9.8%)	18 (22.2%)		8 ( 9.9%)	18 (22.0%)		19 (23.5%)	7 ( 8.5%)	
SUV_max_	5.5 ± 3.5	7.3 ± 3.9	0.003	5.5 ± 3.6	7.3 ± 3.8	0.004	7.0 ± 3.8	5.8 ± 3.8	0.032
NLR	-	-	-	1.6 ± 0.7	4.0 ± 3.8	<0.001	3.8 ± 3.6	1.8 ± 1.7	<0.001
PLR	96.5 ± 32.2	171.5 ± 103.2	<0.001	-	-	-	166.2 ± 104.8	101.7 ± 38.0	<0.001
LMR	4.8 ± 1.8	3.2 ± 1.4	<0.001	4.9 ± 1.9	3.2 ± 1.2	<0.001	-	-	-

SqCC—squamous cell carcinoma; WD—well differentiated; MD—moderately differentiated; PD—poorly differentiated; AJCC—American Joint Committee on Cancer; SUV_max_—maximum standardized uptake value; NLR—neutrophil–lymphocyte ratio; PLR—platelet–lymphocyte ratio; LMR—lymphocyte–monocyte ratio. * Twenty-five patients have missing pathologic T stage data. ^†^ Twenty-six patients have missing pathologic N stage data.

**Table 3:** Univariate and multivariate Cox regression analysis for overall survival in 137 Stage I, II and III non-small cell lung cancer patients.

	Univariate Analysis			Multivariate Analysis		
	HR	95% CI	*p*	HR	95% CI	*p*
Age (reference: <64.3 years old)	3.51	1.16–3.51	0.009	3.11	1.67–5.8	<0.001
Sex (reference: male)	1.45	0.83–2.53	0.2	1.79	0.91–3.52	0.09
Smoking Status (reference: never smoker)	1.25	0.76–2.04	0.4	-	-	-
Histology (reference: non-SqCC)	1.4	0.85–2.30	0.2	-	-	-
Differentiation (reference: WD and MD)	2.34	1.25–4.38	0.006	2.89	1.43–5.83	<0.001
Lymphovascular invasion (reference: absent)	1.51	0.88–2.58	0.10	-	-	-
T stage (reference: 1)						
2	1.03	0.59–1.81	0.003	0.7	0.38–1.29	0.25
3	3.02	1.54–5.91		1.37	0.52–3.63	0.53
4	1.17	0.28–4.99		0.48	0.08–2.73	0.41
N stage * (reference:0)						
1	1.65	0.90–3.05	0.01	1.07	0.37–3.08	0.9
2	2.26	1.25–4.09		2.54	0.30–21.43	0.39
3	3.15	0.43–23.28		1.93	0.12–31.83	0.64
AJCC stage (reference: I)						
Stage II	2.48	1.37–4.50	<0.001	3.41	1.18–9.91	0.02
Stage III	2.86	1.54–5.30		1.52	0.16–14.39	0.72
GLUT1 (reference: negative)	1.96	1.04–3.66	0.036	1.76	0.88–3.53	0.11
GLUT3 (reference: negative)	1.25	0.45–3.44	0.67	-	-	-
FDG-PET SUV_max_ (reference: low)	0.71	0.44–1.16	0.2	-	-	-
NLR (reference: low)	1.59	0.97–2.59	0.06	-	-	-
PLR (reference: low)	1.01	0.62–1.65	0.961	-	-	-
LMR (reference: high)	2.04	1.24–3.34	0.04	2.23	1.28–3.86	<0.001

HR—hazard ratio; CI—confidence interval; SqCC—squamous cell carcinoma; WD—well differentiated; MD—moderately differentiated; AJCC—American Joint Committee on Cancer; SUV_max_—maximum standardized uptake value; NLR—Neutrophil–lymphocyte ratio; PLR—Platelet–lymphocyte ratio; LMR—Lymphocyte–monocyte ratio. * One patient has missing pathologic N stage data.

## Data Availability

Not applicable.
